# Effect of Polysiloxanes on Roughness and Durability of Basalt Fibres–Reinforced Cement Mortar

**DOI:** 10.3390/polym10040420

**Published:** 2018-04-09

**Authors:** Danuta Barnat-Hunek, Grzegorz Łagód, Stanisław Fic, Monika Jarosz-Hadam

**Affiliations:** 1 Faculty of Civil Engineering and Architecture, Lublin University of Technology, Nadbystrzycka 40, 20-618 Lublin, Poland; d.barnat-hunek@pollub.pl (D.B.-H.), s.fic@pollub.pl (S.F.); 2 Faculty of Environmental Engineering, Lublin University of Technology, Nadbystrzycka 40B, 20-618 Lublin, Poland; 3 Faculty of Economics and Engineering, Pope John Paul II State School of Higher Education in Biała Podlaska, Sidorska 95/97, 21-500 Biała Podlaska, Poland; m.jarosz-hadam@dydaktyka.pswbp.pl

**Keywords:** polysiloxanes, mortar, basalt fibre, roughness, surface free energy

## Abstract

The influence of roughness and the way it affects the adhesion properties and surface free energy (SFE) of polysiloxanes hydrophobised basalt fibres–reinforced cement mortars were determined in this article. The physical properties of mortars were investigated in the experimental part, which also explored the impact of hydrophobisation and basalt fibres (BF) addition on SFE, frost resistance, contact angle (CA), and roughness. A device capable of calculating all parameters was used to indicate the surface roughness and 3D topography. Prior to and after conducting surface and weight hydrophobisation, the contact angle of mortars was specified. Subsequently, it was used for carrying out SFE calculation by means of Neumann’s method, enabling us to characterize the adhesion properties and wettability of mortars. The research indicated that the surface roughness was substantially decreased, in turn raising the frost resistance. The corrosion resistance drops when the surface roughness, water absorption, and number of fibres in the mortar increase. The SEM images presenting the structure of polysiloxane coating and mortars were provided.

## 1. Introduction

The basalt fibre (BF), developed in Moscow Research Institute of Glass and Plastic in 1953–1954, is a technologically advanced fibre [1,2]. While analyzing its chemical composition it can be observed that silica constitutes the main component (over 45%), followed by Al_2_O_3_ (approximately 11%–13%) [1,2,3,4]. The diameter of fibres usually ranges from 10 to 20 μm, whereas the length varies from 3 to 120 mm [1]. The production process of basalt fibres consumes less energy and is cheaper than in the case of glass or carbon fibres [5]; additionally, it is not hazardous or carcinogenic [6]. According to Hao L.C. and Yu W.D. the thermal stability of basalt fibres is superior to the one characterizing glass fibres [7]. On the other hand, Moiseev E.A. et al. noted that the crystallization behavior and presence of iron oxide in the production of basalt fibres have an impact on the heat temperature stability [8,9]. The literature studies indicate that basalt fibres are employed in mortars in order to reinforce cement matrices, owing to their properties including high durability, better stability, improved toughness, and ability to resist repeated impact [4]. The basalt fibres in mortars improve the flexural and tensile strength, ductility, as well as cracking energy of cement materials. The professional literature indicates that a sensible addition of basalt fibres amounts to approximately 1–3% (*w*/*w*) of binder [4]. The addition of basalt fibres to the mortar reduces the drying shrinkage and greatly improves the abrasion resistance and the resistance to frost and alkali [10,11]. The content of fibres frequently increases the porosity and absorptivity, which was indicated by the authors in another work [12]. The effect of porosity within the interfacial zone between layers on pull-off adhesion was described. by Sadowski Ł. et al. [13]. The morphology of concrete surface plays an important role in majority of classic physical phenomena, such as electric and thermal conductivity, adhesion, and wettability [14,15]. Sadowski Ł. and Mathia T.G. observed that the morphology of concrete surface depends on aggregate size, material strength, moisture and cleanliness of substrate, and preparation of the surface [14]. The corrosion of elements repaired with concrete is strongly dependent on the appropriate adhesion between layers [16,17].

The content of short basalt fibres causes changes in the topography and roughness of the surface, which is closely connected with the adhesion of these mortars to the substrate, as well as wettability of mortars and adhesion of paints or impregnating preparations. Theoretically, knowing the structure of a rough surface, it is possible to predict the general behaviour of a liquid on it. There are empirical theories pertaining to the wettability of rough surfaces. Usually, it is assumed that if *R_a_* < 0.5 µm, the impact of roughness on the contact angle is insignificant [18]. Certain publications describe the physical mechanisms characterizing this phenomenon [19]. Wenzel [20] noted that a rough surface with hydrophobic film behaved as if it was more hydrophobic in certain spots and more hydrophilic in other. Wenzel also suggests that the surface geometry has a significant impact on the static contact angle [20]. Bico et al. [21] indicated the influence of concrete surface geometry on the adhesion of repair systems. The obtained results indicate that in the case of surface roughness, the occurrence of cracks and loose concrete chunks are important factors that have an impact on the adhesion of repair systems, similarly to, e.g., hydrophobisation.

One of the methods of protecting wettable mortar surfaces is the impregnation of porous construction materials [22,23,24]. Organosilicon compounds are frequently used for the hydrophobisation of construction materials. Silanes and siloxanes are considered one of the most efficient and safest hydrophobising agents. Alkyl potassium silicates, alkoxysilanes, hydrated siloxanes, and siloxanes in hydroxide form are used as silicone hydrophobisers. Alkyl potassium silicates are commercially available only as a highly alkaline (pH = 14) aqueous solution. The remaining compounds are soluble only in organic solvents.

The studies indicated high hydrophobisation efficiency of mortars and lightweight concretes [22,25,26,27]. Silanes, siloxanes, and silicone resins do not differ in respect to the activity, but rather in terms of the molecule size and structure. The pore diameter and surface porosity indicate the possibility of adsorption of a compound and the critical size of a molecule [28]. Certain compounds, including molecular siliconates are characterized by large diameters, which prevent the penetration of the fine-pored structure of some materials, such as cement mortars. Because polysiloxanes agents are often used in the hydrophobisation of building materials, a preparation with large molecules was applied in our research. The chemical composition of monomers is the main factor governing the hydrophobisation efficiency. The alkyl groups (R) regulate the concrete hydrophobisation efficiency, while the alkoxyl groups (OR’) determine the compound reactivity. The type of solvent, i.e., a carrier, is a decisive parameter when it comes to choosing appropriate preparation. The applicable solvents include alcohol, white sprit, and water. However, the alcohol-based preparations do not guarantee achieving the required hydrophobisation efficiency. Therefore, they are not used in civil engineering [12,29].

To the best of our knowledge, basalt-reinforced hydrophobised cement mortars have not been tested in terms of roughness and its influence on wettability, surface free energy (SFE), and frost corrosion. Therefore, determining the relation between interphase roughness of cement mortars as well as wettability and SFE seems necessary because the roughness of cement mortars has a great impact on the type of damage [29]. The physical condition of thin siloxane film in capillaries of porous materials was investigated and analyzed on the basis of mortars reinforced with basalt fibres. The research performed on the surface of cement mortars aimed to indicate a diversified geometrical structure of the considered cement mortars containing basalt fibres, taking into account their surface free energy and frost resistance following hydrophobisation carried out by means of a methyl silicone resin in potassium hydroxide.

## 2. Materials and Methods

The presented research consisted of three stages of examinations: characterisation of cement mortars and water repellent agents, description of the sample preparation and test methods, and determination of the parameters of hydrophobised mortars.

### 2.1. Characteristics of Materials

Two series were prepared, each comprising four mortars with BF. Their compositions per (kg·m^−3^) are presented in Table 1.

Series 1 involves the reference mortars (M0–M1.5) without hydrophobising agent addition, whereas Series 2 (H0–H1.5) includes an addition of cementitious waterproofing material—siloxane. The surface-hydrophobised samples were designated as MR (resin hydrophobised series 1—M) and HR (resin hydrophobised series 2—H). The addition was dosed in the amount of 2% in relation to cement mass. Mortars in each series differed in respect to the BF content. The quantity of BF varied in percentage: 0; 0.5; 1; 1.5%. The water/cement (*w/c*) ratio equal to 0.45 was assumed. The technical parameters of applied Portland cement CEM I 32.5R are presented in Table 2.

**Figure 1:**
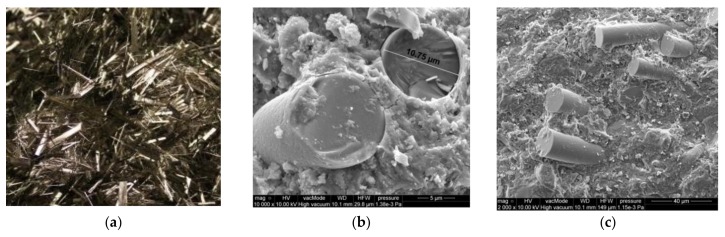
Basalt fibres: (**a**) loose, (**b**) in mortar ×10,000, (**c**) in mortar × 2000.

The characteristics of applied BF are presented in Table 3 [2,3,30]. The employed loose fibres and fibres in mortars were shown in SEM images in Figure 1.

The utilized basalt fibres were about 24 mm in length, with low diameter of 11–18 µm. The fibres were characterized by rough and irregular surface. The constituent minerals of the considered igneous basalt rock include plagioclase: Na(AlSi_3_O_8_)-Ca(Al_2_SiO_8_); pyroxene: XY_2_[(Si, Al)_2_O_6_] (where X—Ca, Mg, Fe^2+^ and Y stands for Fe^3+^, Al, Ti); and olivine: (Fe, Mg)_2_ SiO_4_, which was proven by Kamiya S. et al. [31] in a US Patent.

The classification of waterproofing preparations was carried out on the basis of the authors’ own studies [22,28,32]. Mineral waterproofing grout-siloxane was used in the Series 2. It is a highly cementitious waterproofing admixture of siloxane for mortars with the following properties: strong adhesion to the substrate, water impermeable, resistant to water and frost, resistant to the effects of mechanical and chemical loads. The characteristic data of the product are described in Table 4.

The technical parameters of the applied surface and water hydrophobisation agent are presented in Table 5. The surface hydrophobisation was conducted using macromolecular polysiloxane in potassium hydroxide in an aqueous solvent: R−O−Si−[O−Si]_n_−O−Si−R, where: R—methyl derivative.

### 2.2. Methods

The studies on CEM I 32.5R were conducted in accordance with PN-EN 197-1:2012 standard [33]. Samples with the dimensions of 40 mm × 40 mm × 160 mm were prepared on the basis of EN 196-1:2016-07 standard [34]. All components, except for basalt fibres, were first mixed for two minutes. Afterward, BF were added. Following another two minutes, water was added and mixed for subsequent two minutes. Moulds were filled to the half of their capacity and compacted for one minute on a flow table. Afterward, a second layer of mortars was laid, and the samples were compacted again. The samples were stored under laboratory conditions for 24 h and then removed from the mould and stored in a climatic chamber at the temperature of 23.5 °C and relative humidity of 73.5% for 28 days.

Investigations pertaining to density, as well as volumetric and apparent densities, and open porosity of mortars were carried out in line with PN-EN 1015-10:2001 standard [35]. The research involved six samples with the dimensions of 40 mm × 40 mm × 160 mm. Prior to the investigation, the samples were dried to a constant mass. The flexural strength test was carried out 28 days after moulding, on the basis of EN 1015-11:2001 [36] on three samples of each mortar. The compression strength test was performed on halves of the mortar blocks obtained in the course of flexural strength test (6 samples). The samples were dried at the temperature of 65 °C to a constant weight, weighed, and hydrophobised (by applying the preparation twice using a brush) with the preparation of polysiloxane in potassium hydroxide. The preparation-water ratio was 1:8. Before other investigations were carried out, the samples were seasoned for 7 days under laboratory conditions in order to enable the hydrolytic polycondensation to occur, yielding polysiloxane gel in the subsurface zone of mortars.

The efficiency of hydrophobisation and its influence on the durability of mortars with basalt fibres was evaluated afterwards. Absorptivity of mortars was indicated in accordance with BS 1881-122:2011 standard [37] and EN ISO 7783:2012 standard [38]. The samples were studied in 1 and 14-day intervals in order to determine the impact of moisture on the hydrophobized mortars. The resistance to freezing-thawing (F-T) cycles was determined on the basis of PN-EN 12012:2007 standard [39]. The experiment involved the application of six samples for each batch of mortar with the dimensions 40 mm × 40 mm × 160 mm. The samples were periodically frozen at the temperature of −18 ± 2 °C for at least 4 h and then thawed in water at the temperature of 18 ± 2 °C for a period between two and four hours. Each freeze-thaw (F-T) process was a single experiment cycle. Fifty cycles were conducted instead of the required 25 to prove the increased frost resistance of the examined mortars. After the final cycle was finished, the samples were dried to a constant weight and weighed again to measure the mass lost after determining the frost resistance. The compression strength test of mortars was performed as well.

Determination of surface roughness and 3D topography was conducted in a T8000 RC120-400 device by JENOPTIC, Jena, Germany, similarly to the procedure described in Barnat-Hunek et al. [29]. The measurements were performed using unified GUI (graphical user interface), enabling to calculate all parameters of considered roughness and undulation profiles, as well as evaluation of the geometric characteristics both before and after conducting the surface hydrophobisation. The device has a vertical measuring range of 60 mm and resolution of 50 nm, as well as wavecontour™ digital probing system with digital linear scales in the Z and X-axes. Roughness was measured on inclined or bent surfaces with the resolution of 6 nm in the measurement range of 6 mm. Roughness and contour are evaluated by two separate probing systems. Ten randomly selected fragments of mortar samples were examined, measuring the porosity of a 3.5 × 3.5 mm^2^ area. The measurement time of a single area approximated two hours. The images of surface structure and profilograms obtained directly by using a 3D profilographometer reflected the obtained roughness parameters characterizing the surface of studied mortars reinforced with basalt fibres.

The mortars containing basalt fibres have quite uneven surface, which may be characterized by the following parameters [29,40]:

- *R*_a_—Average Roughness defined as the average deviation of the profile in relation to its mean line and parameter more sensitive to peaks and valleys;
- *R*_pm_—Mean Peak Height defined as the mean peak height from each length of sampling;
- *R*_vm_—Mean Valley Depth as the mean maximum value of valley depth for each length of sampling;
- *R*_p_—Maximum Peak Height as the maximum height of peak within evaluation length;
- *R*_v_—Maximum Valley Depth as the maximum depth observed within the evaluation length;
- *R*_max_—Maximum Peak-to-Valley Height understood as the maximum peak-to-valley height within any of the sampling lengths; *R*_max_ *= R*_v_ *+ R*_p_.

**Figure 2:**
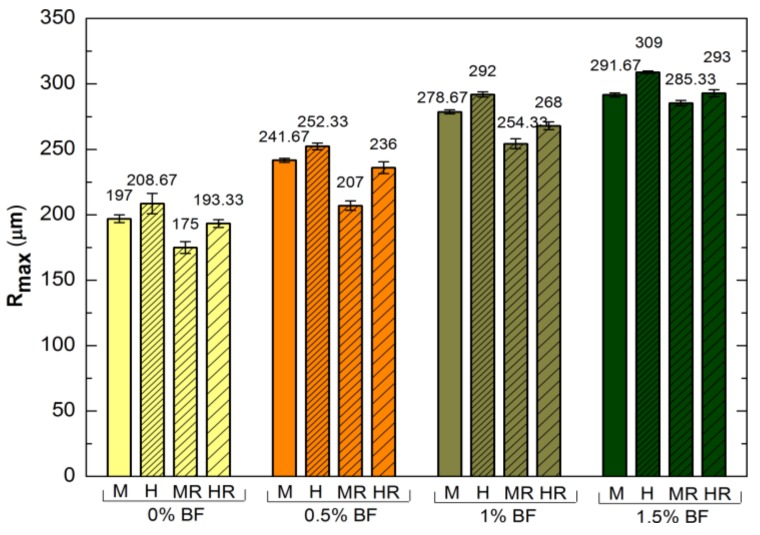
Maximum Peak-to-Valley Height (*R*_max_) of basalt fibres-reinforced cement mortars before and after hydrophobisation with polysiloxanes.

The contact angle (CA) characterizing the liquid drop was measured on a research stand, comprising a goniometer and a camera used for capturing the image of a drop put onto surface of a sample. The analysis of the contact angle was conducted with distilled water. Wetting angles $\theta_{w}$, which correspond to the surface coatings, were measured with a liquid characterized by known total SFE values $\left(\gamma_{w}\right)$. The constant volumes of liquid drops approximated 2 mm^3^ and were applied onto the sample surface by means of a micropipette. The surfaces of samples were corrected smoothed to mitigate the impact of surface roughness. Five drops were applied onto each sample, given the heterogeneity of the material. The measurements were conducted at the temperature approximating 22.5 °C at the moment of applying a drop (Figure 2).

The Neumann model, which constitutes one of the most common methods of calculating SFE, was used in order to determine this parameter [12,22]. The employed equation was as follows [41]:

(1) $$cos\theta_{w}=\left[e^{-0.000125{\left(\gamma_{s-}\gamma_{w}\right)}^{2}}2\sqrt{\frac{\gamma_{s}}{\gamma_{w}}}-1\right]$$

where: $\gamma_{S}$—total SFE, $\gamma_{w}$—SFE of water = 72.8 (mJ·mm^−2^), θw—water contact angle.

Scanning electron microscopy (SEM) (Quanta FEG 250 microscope by FEI, Hillsboro, OR, USA) was employed to determine the morphology, structure of mortars, distribution of polysiloxane gel, and the interfacial transition zone between the cement paste and BF. The samples for SEM studies were glued to a carbon holder by means of carbon glue. Afterwards, they were covered with a 50 nm thick layer of carbon in a coating machine in order to achieve conductivity on the surface of sample.

## 3. Results

### 3.1. Properties of Basalt Fibres–Reinforced Cement Mortar

Table 6 presents both the mechanical and physical properties characterizing the considered mortars prior to being hydrophobized.

### 3.2. Effectiveness of Hydrophobisation of Basalt Fibres–Reinforced Cement Mortar

#### 3.2.1. Roughness and Microstructure of Mortars

The characteristics of roughness obtained for the tested mortars are presented in Table 7.

Figure 2 presents the changes of mortar *R*_max_ with reactive siloxane (Series 2) and the changes following the surface hydrophobisation with polysiloxane in potassium hydroxide.

**Figure 3:**
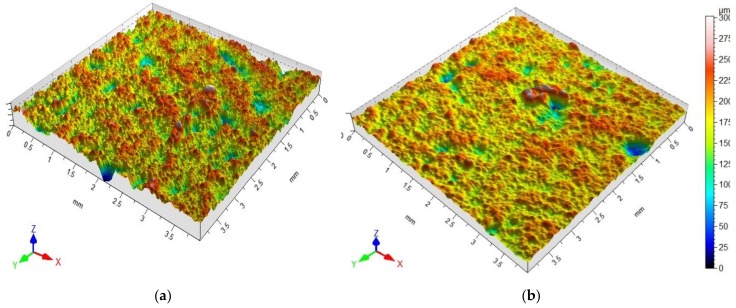
Microroughness and representative profilograms showing the surface of basalt fibres–reinforced cement mortars: (**a**) unmodified M1; (**b**) M1 with polysiloxane coating.

Microroughness and the representative profilograms showing the surface of M1 basalt fibres-reinforced cement mortars—before and after surface modification with polysiloxanes—are presented in Figure 3 a,b.

**Figure 4:**
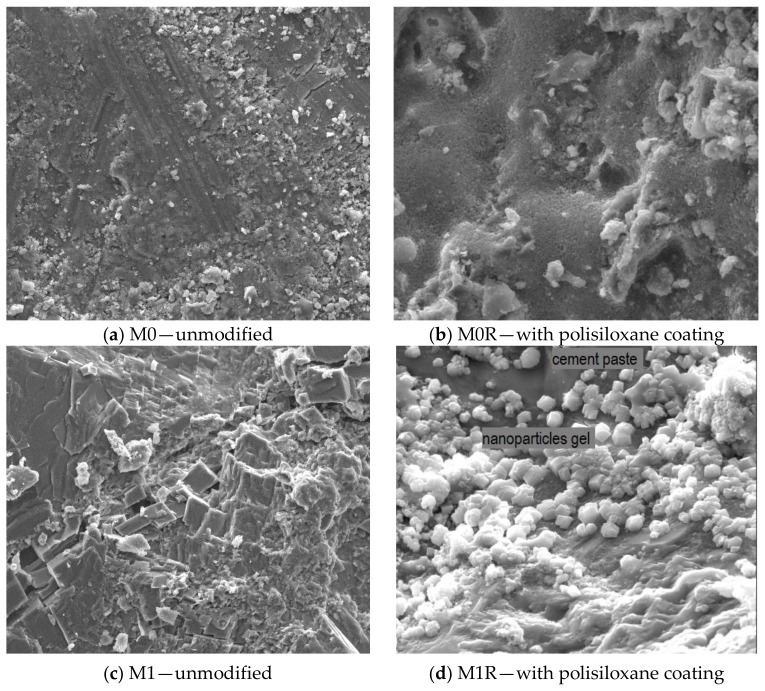
Surface microstructure of unmodified basalt fibres–reinforced cement mortars and the mortars with polysiloxane coating (×4000).

Figure 4 presents the SEM images showing the surface of basalt fibres–reinforced cement mortars before and after hydrophobisation.

**Figure 5:**
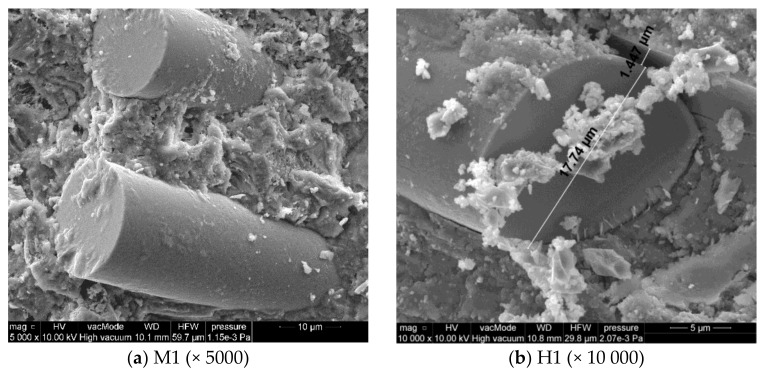

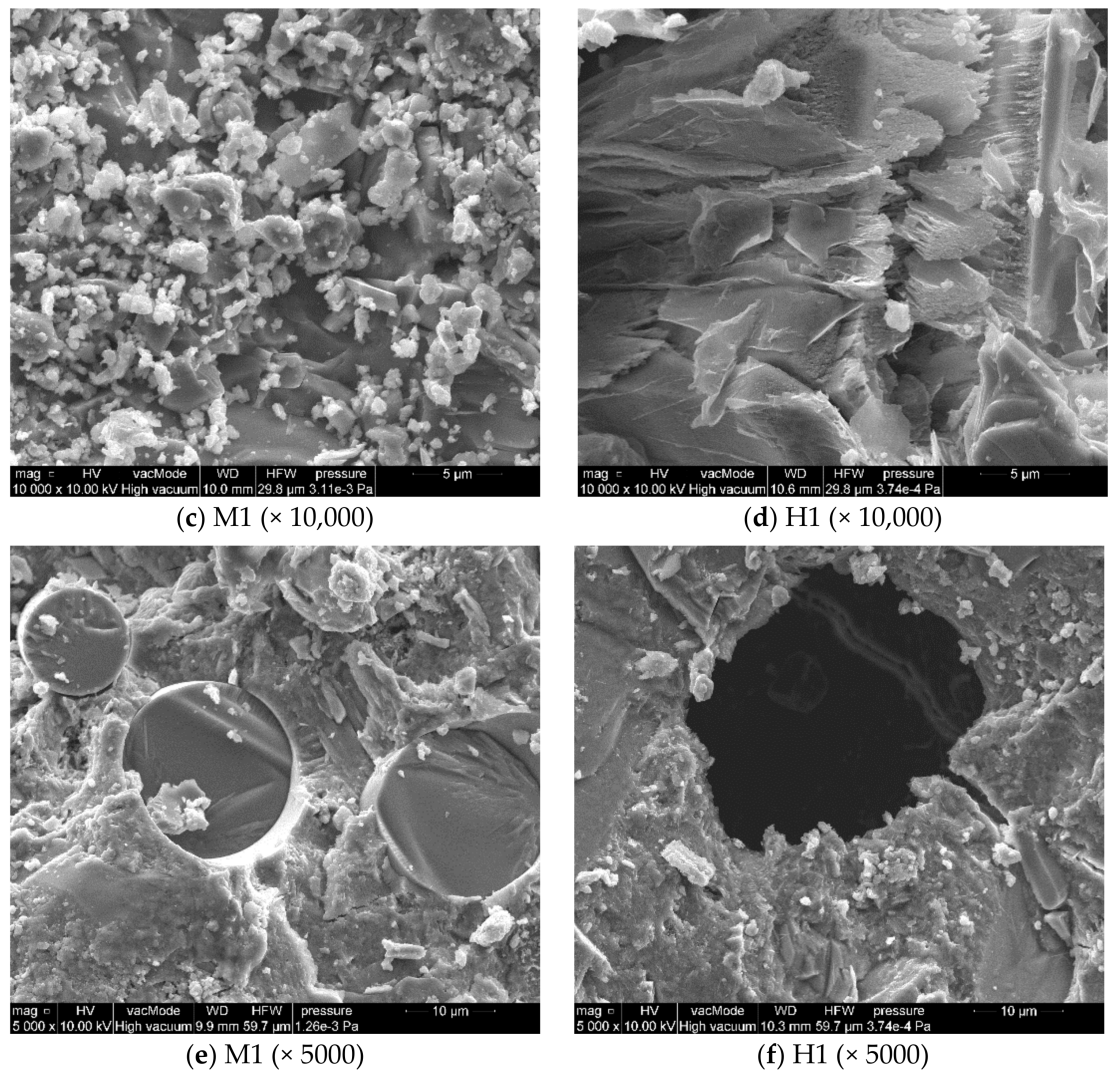
Internal microstructure of basalt fibres–reinforced cement mortars: unmodified (M1) and with siloxane admixture (H1).

Figure 5 presents the microstructure and adhesion of cement paste to BF.

#### 3.2.2. Absorptivity, Wettability, Surface Free Energy (SFE) of Mortars

**Figure 6:**
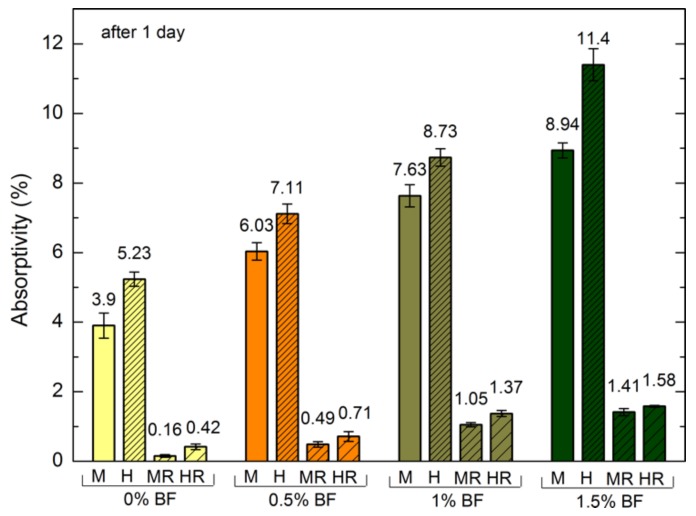

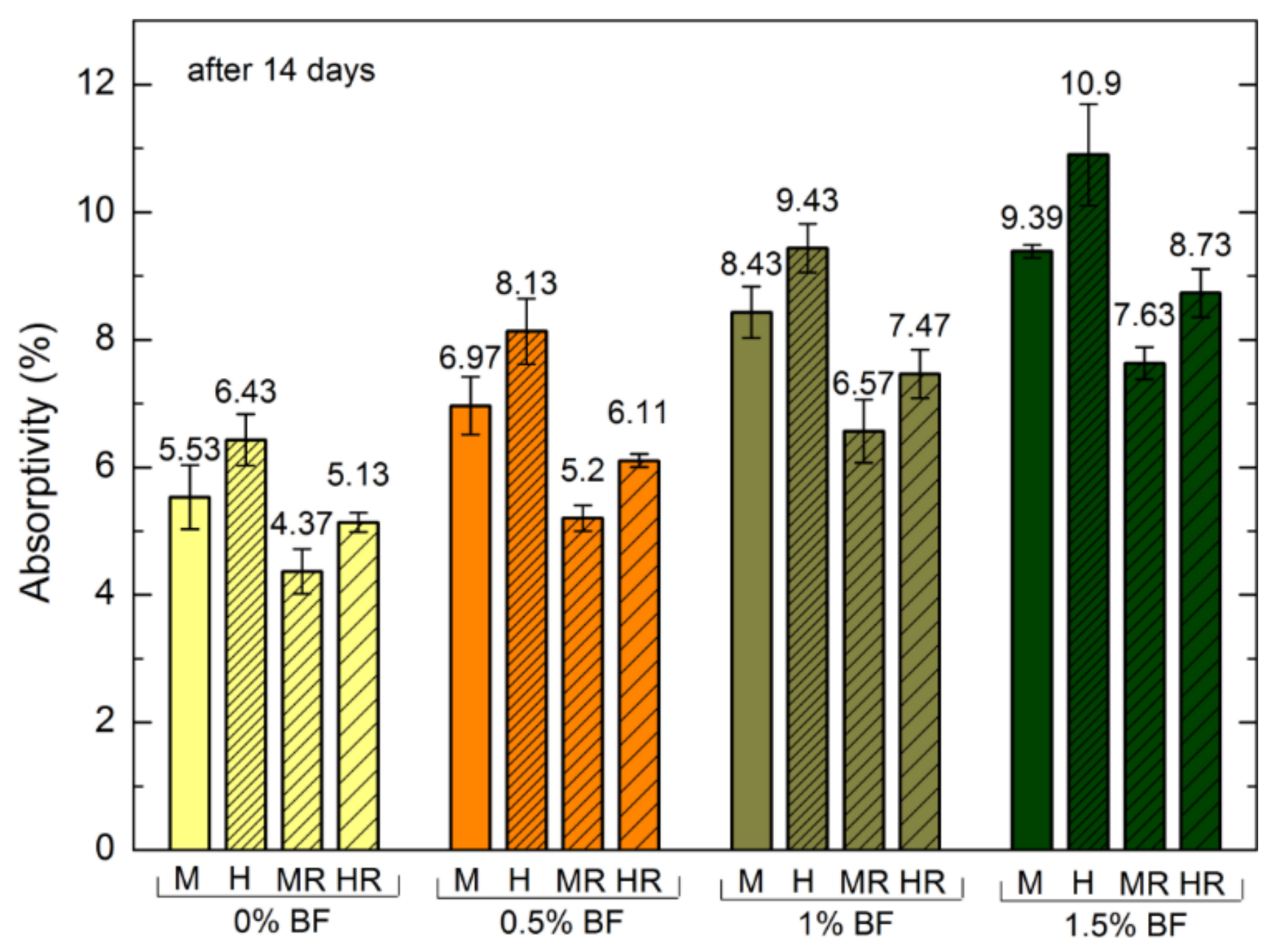
Absorptivity of hydrophobized and reference mortars with BF after one and 14 days.

In order to show the impact of hydrophobisation on the changes in absorptivity in time, an experiment was conducted after one and 14 days. The results are presented in Figure 6.

**Figure 7:**
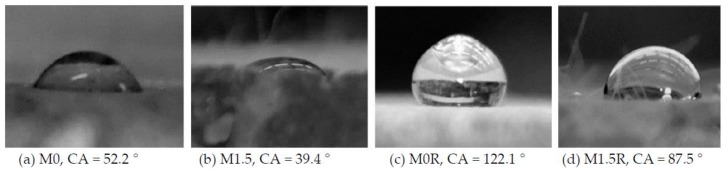
Contact angle of a water drop in mortar with/without BF.

Contact angle (CA) and SFE constitute other parameters defining the wettability of a material [22,29]. Figure 7 presents exemplary photos of CA water measurements of cement mortars. The SFE of all analyzed cement mortars was calculated by means of the Neumann’s method on the basis of CA measurement. Table 8 presents the CA of water measured on each mortar and the SFE calculated on its basis.

#### 3.2.3. Frost Resistance of Mortars

**Figure 8:**
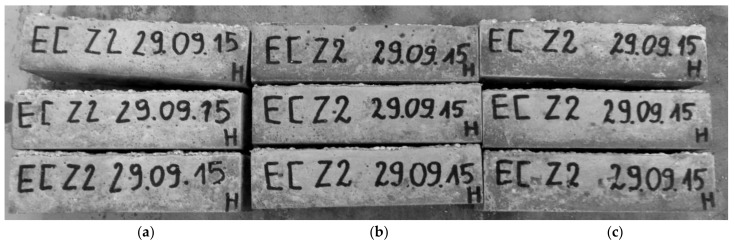
Condition of mortars Series 1 after 50 freeze-thaw (F-T) cycles: (**a**) M1; (**b**) M1R; (**c**) M0R.

**Figure 9:**
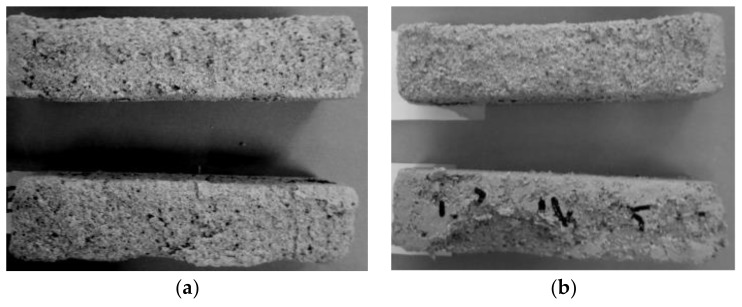
Condition of mortars Series 2 after 50 F-T cycles: (**a**) H0; (**b**) H1.5.

The test used for the evaluation of mortars durability involved investigation of the frost resistance. The condition of samples following a frost resistance test was presented in Figure 8 and Figure 9.

The mass loss and decrease in the compressive strength following 50 F-T cycles was shown in Table 9.

## 4. Discussion

### 4.1. Properties of Basalt Fibres–Reinforced Cement Mortar

Depending on the content of basalt fibres and hydrophobising admixture, mortars are characterized by diversified apparent density ranging from 1741 kg∙m^−^^3^ for the H1.5 mortar with 1.5% basalt fibres and hydrophobising admixture content to 2020 kg∙m^−^^3^ in the case of the standard cement mortar (M0). Porosity varies from 12.9 to 21.1%. The highest porosity was observed in the mortar with hydrophobising admixture and the highest amount of analyzed fibres (H1.5). The drop in the compressive strength resulting from mineral waterproofing grout addition amounts to 24.1% for M0 and 25.5% for M1.5. When 1.5% basalt fibres addition was applied, the flexural strength increased by 32% in Series 1 and 24.6% in Series 2. The presence of basalt fibres caused a great (39%) rise of porosity. The smaller impact of the basalt fibres amount in a mortar can be observed in the results of strength tests. The M0 mortar without fibres or admixture exhibits the highest compressive strength. It can be observed that the strength is determined by the amount of fibres, but mainly by the content of mineral waterproofing grout in the mixtures. Similar conclusions can be formulated while analyzing the results obtained from the examination of physical properties. The strength of samples drops to the value as low as 26%, when mineral waterproofing grout is utilized in mixtures. A similar impact of mineral hydrophobising admixture was confirmed by authors in the studies on other lightweight cement mortars [32]. A grain of cement is covered with polymer film, which hampers or prevents cement hydration, which was described by Łukowski P. [42]. Hence, the decrease in the compressive strength occured. The 5% *w*/*w* admixture to the cement is insufficient to create separate continuous phase in the concrete mixture. In principle, polymer should form a continuous film in the cement binder. The polymer admixture causes the formation of chemical bonds between particular concrete components, having an influence on the changes in microstructure as well as the changes in strength parameters. Unfavourable factors include the bilateral disruption of the polysiloxane gel hardening process in the aqueous environment of cement slurry and hydration of cement in the presence of polymer compound. The presence of water weakens the resin hardening process and thus the final polymer cross-linking reaction forming a film of polysiloxane gel is disrupted. It is likely that this phenomenon occurred with Series 2 cement mortars. In the case of the siloxane admixture utilized in the studies, it had an undesirable influence on the strength of mortars. As far as the lightweight cement mortars with expanded clay aggregate, perlite or zeolite are concerned, the active polycondensation reaction has occurred, forming cross-linked resin which may reinforce the material, which was shown by Barnat-Hunek D. [43]. The porous interfacial zone between the slurry and aggregate was sealed by polymers, which was confirmed in the research conducted by Czarnecki L. and Łukowski P. [44]. Thus, the adhesion of the slurry to aggregate is improved in the contact zone, thus increasing the strength of the entire material, as proven by Shaker F. et al. [45].

Following the analysis involving estimation of the pore structure parameters of the composite using Mercury intrusion porosimeter, thermogravimetric analysis and strength tests, Palou et al. [46] stated that the porosity by crystallization pressure could be enhanced in hardened pastes, which reduce the compression strength. Due to a drop in the strength, Palou et al. recommended that the share of fibres in a mortar should be within the approximate range of 0.1%–1.5% of cement weight [46]. Quattrociocchi et al. [47], who added 1, 3, 6% BF to cement mortars, reported that the dispersion of fibres proved to be more difficult as their content increased in the mixture, which in turn reduced its workability. Therefore, it was necessary to add more water to the mixture, causing a potential increase in porosity. This significant reduction in liquidity was caused by the formation of spatial cross-linked structure due to a random distribution of fibres mixed with the mortar, which was proven in the research carried out by Ma et al. [48].

In our paper, BF improved the flexural strength by about 30%. Dong et al. [49] indicated that the thermal contraction in a cement mortar can be reduced through reinforcement with basalt fibres. The fibres not only increase the flexural strength, but also improve the shock resistance and resistance against thermal contraction cracking. Semenov et al. [50] report that flexural tensile strength increased by 55% in the case of 1% basalt fibres addition, in comparison with the reference mortar samples. Li et al. [51] stated that cement mortars with basalt fibres find practical application in repairs. They observed that 30% of maximum crack width of bent, repaired reinforced concrete elements can be controlled.

### 4.2. Effectiveness of Hydrophobisation of Basalt Fibres–Reinforced Cement Mortar

#### 4.2.1. Roughness and Microstructure of Mortars

The presented study on surface roughness enables to indicate differentiation in the geometrical structure of the surface of both hydrophobized as well as reference materials, in terms of mechanical adhesion. This structure may govern the infiltration of polysiloxanes into rough surface of cement mortars with BF and increase the share of mechanical abrasion, thus influencing the strength of adhesion bonds and the hydrophobisation efficiency.

The analyzed roughness parameters indicate that the content of basalt fibres caused an increase in the average roughness *R*_a_ as well as *R*_vm_ the mean maximum value of valley depth for each length of sampling, *R*_p_ the maximum height of peak within evaluation length and the maximum valley depth—*R*_v_. *R*_max_ is the highest for the mortar with the greatest basalt fibres content and is 32% higher than *R*_max_ of the reference mortar both in Series 1 and 2.

Reactive polysiloxane utilized in Series 2 mixtures increased the average roughness *R_a_* by up to 31% for the standard mortars and by up 13% with the highest share of basalt fibres (H1.5). However, Ra is only a mean value, which does not indicate the largest deviations on the material surface. Therefore, the *R*_max_ values that indicate the highest amplitude being a sum of the largest peak and deepest valley, were analyzed (*R*_p_ + *R*_v_). The *R*_max_ parameter is highest after the application of reactive siloxane admixture for all analyzed cement mortars, regardless of the BF amount. Following surface hydrophobisation, roughness is reduced by 11%–16% in the case of non-modified cement mortars, and by 4%–6% for Series 2 mortars. The mortars with the highest amount of BF are characterized by the greatest roughness, which is 30%–32% higher than *R*_max_ of standard cement mortars. According to Hay et al., impregnation is hindered by a multitude of pores and voids on the material surface, in addition to high roughness. This is particularly apparent when a preparation with high surface tension and viscosity is used, subsequently depositing on the peaks found on a rough surface [52]. A border layer weakening adhesion is formed when bubbles of air become trapped under the thin film of hydrophobising agent [43]. It is likely that the interaction characterizing the substrate and molecules of polymer coating is weakened when roughness is excessive.

The obtained results pertaining to the surface roughness parameters of the considered cement mortars are reflected in the images of surface structure and profilograms, obtained directly by means of a 3D profilografometer. The representative surface profilograms (Figure 3) of basalt fibres-reinforced cement mortars present visible differences in the microroughness of cement mortars before and after the application of polysiloxane film. Large polysiloxane gel molecules partially fill the pores of rough surface of mortars with BF addition, which was clearly presented in SEM images (Figure 4b,d). In the case of M0R mortars without basalt fibres, a thick, tight film of siloxane gel was formed. This situation is undesirable, because higher hydrophobisation efficiency is obtained when a thin film of polysiloxanes is formed in the material structure. This film slightly fills the pores of porous material forming a thin hydrophobic layer, which is more resistant to damage, cracks, and scratches, resulting from, e.g., frost corrosion [22,29,43]. In the case of the cement mortars with basalt fibres (Figure 4d), crystallization of polysiloxane gel phase, which loosely covers the surface of cement mortars, was clearly observed. Due to increased porosity, a thick, tight film was not formed.

Figure 5a,b,e depicts the adhesion of cement paste to BF. It can be observed that in the case of the mortar without siloxane admixture, the adhesion of slurry to fibres is very good. C–S–H phases have clearly crystallized (Figure 5a,e). In H1 mortar with hydrophobising admixture, characterized by higher porosity, 1.5 µm-wide cracks appear between fibres and slurry. Loose contact zone between BF and slurry causes a reduction in compression strength, which was described in Section 4.1.

According to [46], in the earlier period of hydrothermal hardening, hydrated products such as C–S–H were formed in the course of a chemical process. Due to the higher crystallization pressure than in the case of non-modified mortars, the porosity of cement mortars increases (Figure 5f), simultaneously reducing the compression strength. According to Palou et al. [46], a partial thermal decomposition of C_3_AH_6_ to C_3_AH_1.5_ occurs under high pressure in the later stage of the hardening process, resulting in the saturation of a sample with carbon dioxide. Additionally, the content of siloxane admixture slows down the cement hydration, affecting the quality and type of produced phases, which is illustrated, e.g., in Figure 5c,d. Figure 5c presents the C–S–H phases and crystallized ettringite. On the other hand, there are no visibly crystallized ettringite lumps in Figure 5 because they are only found in insignificant amounts. The figure shows the lamellar rather than spherical structure of phases, in contrast to typical cement mortar. This indicates that both BF and siloxane addition caused changes in the structure of mortars, which affects their physical and strength parameters, e.g., high porosity, as shown in Figure 5f.

#### 4.2.2. Absorptivity, Wettability, SFE of Mortars

The highest absorptivity is exhibited by H1.5 (Figure 6) mortar with siloxane admixture which has the highest porosity. In the first day of the experiment, the influence of surface hydrophobisation with polysiloxanes is the most evident. The absorptivity of standard mortars with this preparation was 95% and 92% lower for M0R and H0R, respectively. In the case of mortars with the highest BF content, this difference amounts to 84% and 86% for M1.5R and H1.5R, respectively. After 14 days of research, the differences between the hydrophobized and standard cement mortars were significantly lower and equalled 21% for M0R and 18% for M1.5R.

The obtained results indicate that the type of mortar governs the values of CA. The CA measurements showed that the contact angles of the hydrophobized cement mortars are significantly higher than in standard mortars (by up to 57%). Conversely, the lowest contact angle value was obtained in the case of H1.5 mortar with 1.5% BF content and reactive siloxanes addition. The contact angle drops as the fibres content increases, which is connected with higher porosity and absorptivity of mortars.

The greatest contact angle was observed for M0 mortars, both prior to and following hydrophobisation. In this case, the polysiloxane film changed the adhesion parameters by 57%. Hydrophobisation reduced CA by 9.6% and 23% for H0 and H1.5, respectively. As the content of BF increased, CA exhibited a drop from 7% to 24.5% in Series 1 and from 13.7% to 35% in Series 2.

SFE in the case of all cement mortars before surface hydrophobisation is relatively high and indicates good hydrophilicity of mortars (CA < 90°). Hydrophobisation with polysiloxanes greatly reduced adhesion to mortar surface, which is connected with higher resistance to corrosive liquid infiltration, e.g., salty solutions. Even in the case of H1.5 mortars characterized by highest absorptivity, hydrophobisation turned out to be successful and reduced the negative impact of basalt fibres on wettability, because SFE of this mortar following hydrophobisation (37 mJ·m^−2^) is still 29.5% lower than the one of M0 reference mortars without basalt fibres.

The characteristics of surface properties, especially SFE, are considered important parameters required for understanding the mechanisms of surface phenomena [12,22,43]. SFE is the result of attraction between the molecules in the surface layer and volume of a material [43]. The literature includes papers on applying thin silane films on various substrates in order to modify their final wettability [12,22,29,53]. On the basis of the literature data on SFE of hydrophobized materials, it can be stated that there are few such studies, which was also observed by Cappalletti G. et al. [53]. The authors of [53] examined nine different types of hydrophobic films based on silanes, siloxanes, chlorosilanes, and others. In the majority of cases, the obtained SFE values did not exceed 42 mJ·m^−2^; for octadecyltrichlorosilane were lower than 23.5 mJ·m^−2^. CA over 120° can be obtained through chemical modification of surface with siloxanes. The studies on SFE of silane and polisiloxane films were described, i.a. in the papers by Król P. et al. [54], Courard L. et al. [55], and Selvakumar N. et al. [56]. The paper [54] reports that SFE of silanes did not exceed 23 mJ·m^−2^, and approximately 42 mJ·m^−2^ in the case of triethoxysilanes. Polymer films reached SFE in the range of 21 to 45 mJ·m^−2^. In our work, similar and slightly lower SFE values were obtained for polysiloxane films in aqueous solvent, ranging from 10.53 to 37 mJ·m^−2^ depending on the content of BF and hydrophobising admixture. This indicates a very good hydrophobisation efficiency, especially in terms of protection against water and frost corrosion, which was described in Section 4.2.3.

**Figure 10:**
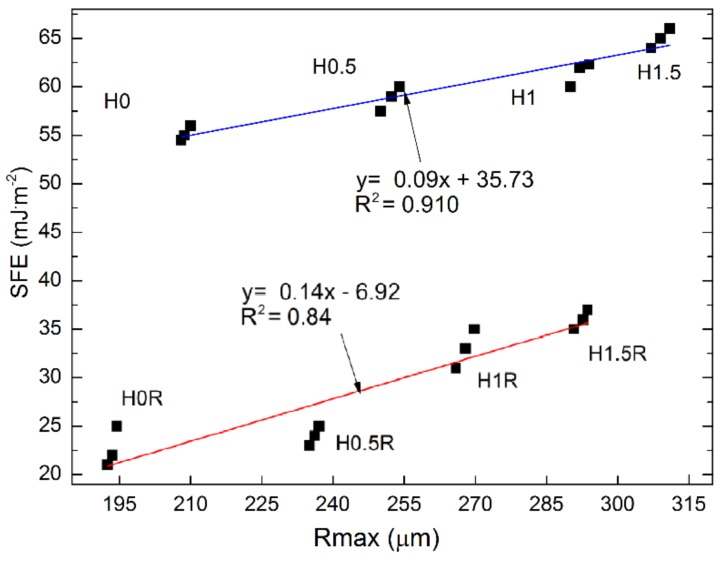
Correlation between roughness and surface free energy (SFE) of mortars with basalt fibres Series 2: blue line—unmodified; red line—hydrophobized surface.

It can be observed that roughness as well as SFE, and consequently wettability of the mortar surface increase along with the content of basalt fibres and siloxane admixture. The polysiloxane film reduces these parameters. Linear dependencies were observed between roughness and SFE; a regression model with a single variable was employed (Figure 10). 

These correlations mainly stem from changes in the porosity of cement mortars depending on their composition and the applied preparation or hydrophobising admixture. This is because porosity is strictly connected with absorptivity, contact angle and SFE. Porosity is also connected with the compression strength. Figure 10 indicates that porosity drops as a result of hydrophobisation, however SFE mainly depends on the chemical changes occurring on the surface of material in the course of polysiloxane film application, not only on roughness. Despite similar roughness of the cement mortars, SFE is much lower for the surfaces covered with polysiloxane film. Siloxanes are capable of forming durable spatial cross-links. Owing to their chemical similarity, the hydrophobic substance forms lasting and resistant bonds with silanol groups present in hydrophilic construction materials after the contact with the mineral surface [43]. During the application of silicones on the surface of material, they bind their inorganic part Si–O–Si with the mineral substrate, directing non-wettable organic groups R outwards, resulting in a significant reduction of SFE value. 

#### 4.2.3. Frost resistance

The frost resistance test did not exhibit significant differences in the behaviour of the cement mortars subjected to the F-T cycles. The hydrophobized samples (Figure 8b,c) showed virtually no damage, similarly to the samples of M1 mortar without hydrophobisation, which were characterized by small chippings on the surface (Figure 8a). In the case of the mortars with reactive siloxane admixture, more serious damage of samples occurred, as presented in Figure 9. Loosening, flaking and losses were observed in the samples.

The greatest mass loss corresponds to H1.5 mortar and amounts to 8.3%, which is over 10 times greater value than the mortar with the same BF content, but without siloxanes (M1.5). Siloxane admixture dramatically lowered the frost resistance of the mortar. The greatest reduction in strength after 50 F-T cycles was observed for this cement mortar, equalling as high as 24.3%, whereas the mortar without siloxanes experienced only a 2.5% reduction. Such situation has occurred because the hardening process of the polysiloxane gel was disrupted in the aqueous environment of cement slurry and hydration of cement in the presence of a polymer compound, which was mentioned in the Section 4.1 and SEM analyses. The admixture had a negative impact on the strength and frost resistance of the cement mortars with BF. However, the application of polysiloxanes as a protective film yielded the expected results. The mass loss of the cement mortars with 1.5% BF dropped almost three-fold, whereas the compression strength reduction decreased almost by the factor of five. Jiang et al. [10] showed that the cement mortars with basalt fibres ale characterised by lower early workability, better flexural strength, abrasion resistance, water permeability, but also a greater mass loss resulting from freezing and thawing, due to higher porosity, which was also indicated in our studies.

One of the principal parameters having an influence on the hydrophobisation efficiency is the adhesion of siloxane films to the substrate. During freezing and thawing, resulting from the infiltration of water through a polysiloxane gel film, delamination may occur at the contact spot between the material and film. No such situation occurred in the analyzed cement mortars with BF. All of the analyzed mortars have successfully protected the samples against the effect of frost. However, there are papers, including the authors’ own works, which indicate that molecules of polysiloxane gel covered concrete too tightly, resulting in excessive ice crystallization pressure, which severely damaged the lightweight aggregate and the contact spot between the aggregate and cement paste [43]. Similar observations related to the high hydrophobisation efficiency were made in the work by Barnat-Hunek D. [43] concerning hydrophobized UHPC concretes with the addition of steel and polypropylene fibres. The concrete with steel fibres is especially vulnerable to frost corrosion. The application of methyl-silicone resins, particularly alkyl-alkoxy-siloxanes, has greatly increased the resistance of UHPC to F-T cycles. Even after 180 F-T cycles, in majority of cases the CA value was still greater than 100°, which indicates good hydrophobicity of concrete surface.

The corrosion resistance characterizing cement mortars is indirectly dependent on the frost resistance.

Frost and salt resistance are considered important elements for the assessment of concrete durability. The transition of water from the liquid to solid phase increases its volume by approximately 9%. When the pores in concrete are almost completely saturated, freezing may cause serious cracks and damage of concrete.

**Figure 11:**
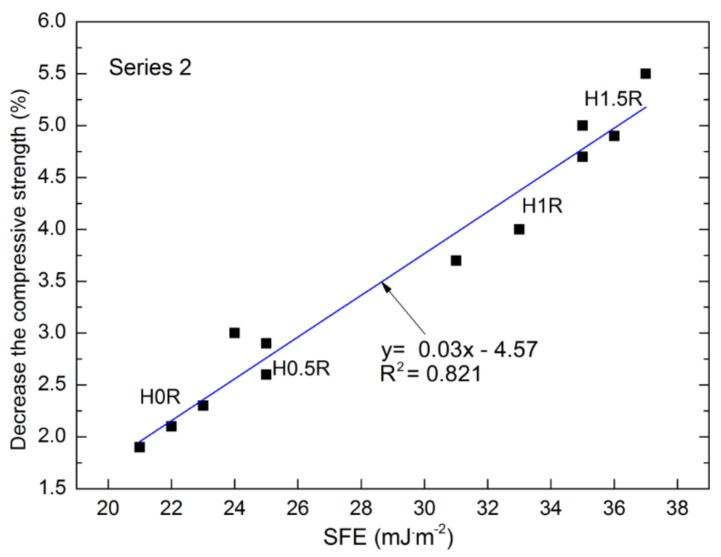
Correlation between SFE and frost resistance of mortars with basalt fibres.

Due to a strong correlation between roughness and SFE, the authors intended to prove that a correlation exists between the variable parameter connected with moisture, adhesion properties (SFE), and frost resistance. In this case, a drop in the strength of cement mortars following 50 F-T cycles corresponded to frost resistance. This parameter, expressed as a percentage of compression strength reduction, depends on SFE. This dependency can be described by means of a linear function (Figure 11).

The correlations described by the formula y=0.03x−4.57 indicate a good coefficient of determination R^2^ = 0.821. Taking into account the data presented in Figure 11 and other obtained results, it can be concluded that both the moisture parameters, as well as adhesion properties (SFE) are closely connected with frost resistance. The lower the wettability and SFE, the higher the frost resistance. Protection of the cement mortars with BF against moisture, provided through surface hydrophobisation, has greatly increased frost resistance.

## 5. Conclusions

This work analyzed the physical properties as well as the microstructure characterizing the cement mortars with basalt fibres subjected to hydrophobisation.

On the basis of the analysis of research, the following conclusions were drawn:

1. The addition of BF has a negative impact on compression strength and frost resistance. However, it improves the flexural strength of the analyzed mortars with and without siloxane addition by 24% and 32%, respectively.
2. The BF addition caused changes in the structure of cement mortars and altered the roughness parameters. Along with the increase of basalt fibres, porosity increases by up to 32%.
3. Reactive polysiloxane applied in the Series 2 mixtures increased the average roughness by up to 31% for the standard mortars and by up 13% with the highest share of basalt fibres (H1.5). The polymer admixture causes creation of chemical bonds between particular concrete components, influencing the changes in the microstructure and the strength parameters. The hardening process of polysiloxane gel in the aqueous environment of cement slurry and cement hydration in the presence of polymer compound are disrupted. The presence of water weakens the resin hardening process, and the final polymer cross-linking reaction yielding a film of polysiloxane gel is disrupted.
4. A linear reaction between the roughness and adhesion properties described by SFE was presented. The highest hydrophobicity was obtained in the case of the reference M0R mortar with SFE equal to 10.53 mJ·m^−2^.
5. The investigation pertaining to the roughness following hydrophobisation, as well as the produced 3D profilograms, confirmed the influence of polysiloxane film on roughness reduction. The cement mortars without the polysiloxane film are characterized by four times greater adhesion, which is connected with the durability of mortars. Following hydrophobisation, the frost resistance of the mortars with 1.5% basalt fibres addition dropped almost three-fold, whereas the reduction in the compression strength decreased almost by a factor of five.
6. Due to the negative impact of siloxane admixture in the cement mortars with basalt fibres additionon the frost resistance, compression strength, and adhesion of cement paste to fibres, its application in this type of mortars is not recommended.
7. Due to a reduction in wettability and increase in frost resistance, performing the surface hydrophobisation by means of polysiloxanes is encouraged in order to improve the durability of cement mortars with basalt fibres.

## Figures and Tables

**Table 1:** Composition of the mortars with basalt fibres.

Materials	Unit	Series 1				Series 2			
		M0	M0.5	M1	M1.5	H0	H0.5	H1	H1.5
Portland cement I 32.5R	(kg∙m^−3^) (kg∙m^−3^) (kg∙m^−3^) (kg∙m^−3^) (kg∙m^−3^)	280	280	280	280	274.4	274.4	274.4	274.4
Quartz sand < 2 mm		1483	1483	1483	1483	1483	1483	1483	1483
Water		126	126	126	126	126	126	126	126
Hydrophobic admixture		-	-	-	-	5.49	5.49	5.49	5.49
Basalt fiber 12–18 µm		-	13.2	26.4	39.6	-	13.2	26.4	39.6
Basalt fiber 12–18 µm	(%)	0	0.5	1	1.5	0	0.5	1	1.5

**Table 2:** Technical parameters of Portland cement CEM I 32.5R [22,29].

Parameters	Unit	Value	Parameters	Unit	Value
Specific surface	(cm^2^∙g^−1^)	3985	Density	(g∙cm^−3^)	3.05
Initial setting time	(min)	248	Volume stability	(mm)	< 10
Loss on ignition by weight cement	(%)	5.0	SO_3_ content	(%)	2.798
Compressive strength	(MPa)		Cl content	(%)	0.066
after 2 days		17.6	Cr(VI) diss. content	(ppm)	0.26
after 28 days		43.2	Na_2_O_eq_ content	(%)	0.78

**Table 3:** Parameters of basalt fibres [2,3,30].

Parameters	Unit	Value
Hardness on the Mosh’ scale	(-)	8.5
Elongation to fracture	(%)	2.4–3.1
Softening temperature	(°C)	960
Modulus of elasticity	(GPa)	89–110
Tensile strength	(MPa)	2800–4500
Thermal conductivity	(W∙m^−1^∙K^−1^)	1.67
Moisture absorption	(%)	<0.1
Coefficient of linear thermal expansion	(K^−1^)	5.5 × 10^−7^
Specific heat capacity	(kJ∙kg^−1^∙K^−1^)	0.86
Constant operating temperature	(°C)	680
Melting temperature	(°C)	1450
Operating temperature range	(°C)	–260 to +750

**Table 4:** Technical parameters of mineral waterproofing grout [32].

Parameters	Unit	Value
Water absorption coefficient *w_24_*	(kg∙m^−2^∙h^−0.5^)	<0.1
Water requirements	(%)	20–21
Water vapour diffusion *δ*	(g∙m^−^^1^·h^−^^1^·hPa^−^^1^)	<200
Compressive strength after 28 days	(MPa)	30
Tensile bending strength after 28 days	(MPa)	6
Working time	(min)	60

**Table 5:** Technical parameters of polysiloxane and water.

Parameters	Unit	Polysiloxane	Water
Density	(g∙cm^−3^)	1.03	0.99
Viscosity *η*	(Pa∙s)	0.98 × 10^−3^	0.89 × 10^−3^
Surface tension *γ*	(N∙m^−1^)	77.24 × 10^−3^	72 × 10^−3^
Surface tension-to-viscosity ratio *η/γ*	(-)	78.82	80.90

**Table 6:** Technical parameters of non-hydrophobised basalt fibres–reinforced cement mortar.

Parameters	Unit	Series 1				Series 2			
		M0	M0.5	M1	M1.5	H0	H0.5	H1	H1.5
Apparent density *ρ*_a_	(kg∙m^−3^)	2020	1956	1941	1823	1952	1892	1823	1741
Density *ρ*	(kg∙m^−3^)	2320	2301	2287	2245	2300	2265	2230	2207
Total porosity *P*	(%)	12.9	14.9	15.2	18.7	15.1	16.5	18.3	21.1
Open porosity *P*_o_	(%)	5.3	7.5	9.2	11.4	8.8	9.9	10.4	12.8
Compressive strength *f*_c_	(MPa)	9.53	9.01	8.31	7.63	7.23	7.06	6.41	5.68
Flexural tensile strength *f*_m_	(MPa)	6.10	6.74	7.87	8.96	5.32	5.98	6.47	7.06

**Table 7:** Roughness characteristics of basalt fibres–reinforced cement mortars.

Type of mortars		Mortar roughness characteristics (µm)					
		*R*_a_	*R*_vm_	*R*_pm_	*R*_p_	*R*_v_	*R*_max_
Series 1	M0	13.3	102	42	52	145	197
	M0.5	16.2	140	70	78.7	163	241.7
	M1	18.4	167	75	90	188.7	278.7
	M1.5	22.3	179	84	95.7	196	291.7
Series 2	H0	19.3	111	50	58.7	151	208.7
	H0.5	19.3	152	79	83.3	169	252.3
	H1	22.9	179	82	99	193	292
	H1.5	25.7	193	92	108	201	309

**Table 8:** Contact angle (CA) and surface free energy (SFE) of basalt fibres–reinforced cement mortars.

Parameters	Unit	Series 1				Series 2			
		M0	M0.5	M1	M1.5	H0	H0.5	H1	H1.5
CA before hydrophobisation	(°)	52.2	48.2	41.5	39.4	47.2	40.7	39.1	30.4
CA after hydrophobisation	(°)	122.1	114.1	94.7	87.5	100.8	98.7	82.5	77.6
SFE before hydrophobisation	(mJ·m^−2^)	52.49	54.81	58.56	59.70	55.38	59.0	59.86	64.29
SFE after hydrophobisation	(mJ·m^−2^)	10.53	14.78	26.32	30.81	22.58	23.86	33.93	37.0

**Table 9:** Mass loss and decrease in the compressive strength of basalt fibres–reinforced cement mortars after 50 F-T cycles.

Parameters	Unit	Series 1				Series 2			
		M0	M0.5	M1	M1.5	H0	H0.5	H1	H1.5
Mass loss before hydrophobisation	(%)	0.3	0.5	0.6	0.8	2.1	2.8	5.3	8.3
Mass loss after hydrophobisation	(%)	0.01	0.1	0.2	0.24	0.7	1.2	2.4	2.7
Decrease the compressive strength before hydrophobisation	(%)	-	1.1	2.3	2.5	4.3	6.7	13.2	24.3
Decrease the compressive strength after hydrophobisation	(%)	-	0.4	0.9	1.1	2.1	3.2	4.5	5.2
